# Rheumatoid arthritis and risks of postoperative adverse events after posterior lumbar interbody fusion for lumbar degenerative diseases: a retrospective cohort study with matched controls

**DOI:** 10.3389/fsurg.2026.1928776

**Published:** 2026-09-18

**Authors:** Zhiyuan Guo, Xiao Liang, Qingtao Liu, Puxin Yang, Yachong Huo, Jiaxin Xu, Hui Wang, Xianzhong Meng

**Affiliations:** 1 Department of Spinal Surgery, The Third Hospital of Hebei Medical University, Shijiazhuang, China; 2 Department of Orthopedics, Cangzhou Central Hospital, Cangzhou, China

**Keywords:** adjacent segment disease, lumbar degenerative disease, osteoporosis, posterior lumbar interbody fusion, postoperative complication, rheumatoid arthritis

## Abstract

**Objective:**

To explore the correlation between rheumatoid arthritis (RA) and postoperative complications, long-term pain relief and functional recovery in patients undergoing posterior lumbar interbody fusion (PLIF) for lumbar degenerative diseases.

**Methods:**

A single-center 1:3 matched retrospective cohort study was conducted. From January 2015 to September 2020, 32 PLIF patients with confirmed RA were included in the exposed group, and 96 non-RA (NRA) PLIF patients were matched by age, gender, body mass index and single/double fusion segment. Binary logistic regression was performed to identify independent risk factors for complications, and unadjusted odds ratios (OR), relative risks (RR) with 95% confidence interval were calculated for each adverse event. All patients completed ≥2-year clinical and imaging follow-up.

**Results:**

All patients acquired immediate short-term postoperative pain reduction; however, RA group exhibited progressive recurrent back and leg pain during long-term follow-up. The postoperative and final follow-up visual analog scale for back and leg pain were significantly higher in the RA group than in the NRA group (*P* < 0.05), and the Oswestry Disability Index score at the last follow-up was also poorer (vs. NRA, *P* < 0.05). The postoperative C reactive protein level was markedly elevated in RA patients (vs. NRA, *P* < 0.05). RA group presented lower L1 Hounsfield Unit (HU) values and a higher incidence of osteoporosis (25.0% vs. 9.38%, vs. NRA, *P* < 0.05). Univariate analysis with unadjusted OR/RR demonstrated that RA was significantly associated with higher crude risks of total complications, wound infection, ASD and revision surgery.

**Conclusion:**

RA is correlated with significantly elevated risks of multiple adverse events following PLIF for lumbar degenerative diseases.

## Introduction

Rheumatoid arthritis (RA) is a chronic autoimmune rheumatic disease characterized by progressive invasive synovitis and bone destruction (1). RA affects approximately 1%–2% of the general adult population, and the prevalence rises to 2.3% among adults aged over 60 years (2, 3). The persistent inflammatory state of RA can destroy intervertebral spaces and facet joints, leading to spinal malalignment, spinal instability and lumbar spinal stenosis (4, 5). Cervical spine lesions are the most common spinal manifestations of RA (6), while lumbar spine involvement only occurs in 3%–5% of RA patients (7, 8).

Accumulated evidence has demonstrated that RA substantially elevates surgical risks for patients receiving orthopedic procedures (9, 10). In total knee arthroplasty (TKA), RA patients have higher risks of postoperative infection, wound dehiscence and revision surgery compared with patients with osteoarthritis (OA) (11). Furthermore, RA patients face an increased 90-day readmission rate due to pneumonia and local infection after TKA. For total hip arthroplasty (THA), RA is correlated with higher rates of prosthesis dislocation, revision, infection, VTE and even mortality (12). As for cervical spine surgery, RA patients exhibit a markedly higher complication rate than ordinary spine surgery patients. The wound infection rate can reach 25% in cases with internal fixation, and the perioperative mortality is up to 10% among RA patients (13). Moreover, fixation failure, subluxation, pseudarthrosis and adjacent segment disease (ASD) are also common complications after upper cervical spine surgery in this population (14).

RA has been reported to elevate infection, thromboembolism and revision risks in total joint arthroplasty and cervical spine fusion (15, 16), while few studies systematically report perioperative and long-term posterior lumbar interbody fusion (PLIF) outcomes in RA patients with lumbar degenerative lesions. This single-center 1:3 matched retrospective cohort study compared perioperative indicators, pain/functional recovery, radiological parameters and postoperative complications between RA and matched NRA PLIF patients, aiming to provide quantitative clinical evidence for preoperative risk assessment and targeted management of RA patients undergoing lumbar fusion.

## Patients and methods

### Statement of ethical approval

This retrospective secondary analysis using historical clinical and imaging data collected from 2015 to 2020 was retrospectively approved by the Institutional Review Board of the Third Hospital of Hebei Medical University (approval No. KS2022-040-1). Per institutional regulatory standards, retrospective ethics approval covers research utilizing fully de-identified archived patient data. As dual compliance assurance, all participants had signed a routine general written informed consent at admission, which authorized the use of their anonymized clinical records for retrospective academic research. All data extraction and analytical procedures performed in this study strictly complied with the principles outlined in the Declaration of Helsinki.

### Patient population

This was a single-center retrospective case-control study. All enrolled patients had failed standardized conservative treatment and met the surgical indications for PLIF.

Inclusion criteria: (i) Patients diagnosed with RA who met the surgical indications for lumbar degenerative diseases; (ii) Patients underwent PLIF or PLIF combined with posterolateral fusion, and all operations were performed by a single senior orthopedic surgeon; (iii) Complete preoperative lumbar x-ray, computed tomography (CT) and magnetic resonance imaging (MRI) data were available; (iiii) The minimum follow-up duration was 2 years. All included RA patients fulfilled the 2010 ACR/EULAR rheumatoid arthritis classification criteria, with seropositive rheumatoid factor (RF) or anti-cyclic citrullinated peptide (anti-CCP) antibody plus persistent inflammatory peripheral synovitis (17).

Exclusion criteria: (i) Patients combined with spinal tumors or primary spinal infection; (ii) Patients with systemic active infection before surgery; (iii) Patients with hematological diseases, severe anemia or coagulation disorders.

From January 2015 to September 2020, a total of 89 RA patients underwent PLIF in our hospital. After screening according to the inclusion and exclusion criteria, 32 eligible RA patients were finally included in the study group. Follow-up was conducted via outpatient reexamination and telephone interviews to record new-onset symptoms and complications during the follow-up period.

### Case matching

Considering the low incidence of lumbar surgery in RA patients, a 1:3 matched case-control design was adopted. Each RA case was matched with 3 non-RA (NRA) controls based on four confounding factors: age (difference ≤5 years), gender, body mass index (BMI) (difference ≤2 kg/m^2^), and fusion segment (single-level/double-level fusion). A total of 32 RA patients and 96 matched NRA patients were finally enrolled. This design is defined as a matched retrospective cohort study, rather than a case-control study, as we enrolled all patients who received PLIF surgery and grouped them by RA exposure status with matched non-exposed controls to balance baseline confounders.

### Clinical assessment

Demographic data including age, gender, BMI, Steinbroker classification (Grade I–IV, used to evaluate RA and disease severity; higher grades indicate more severe conditions) and disease duration of RA were collected. Perioperative parameters, including operative duration, intraoperative blood loss, bed rest time and hospital stay, were recorded in detail. Clinical efficacy was evaluated using visual analog scale (VAS) for back pain and leg pain, as well as the Oswestry Disability Index (ODI).

We extracted complete long-term antirheumatic medication records of all RA patients from electronic medical systems and archived paper charts. The recorded regimens covered conventional synthetic DMARDs, TNF-α inhibitors, IL-6 inhibitors, abatacept, rituximab and oral JAK inhibitors. Daily oral glucocorticoid exposure was further stratified by prednisolone equivalent dose (<5 mg/d or ≥5 mg/d). We also fully documented the institutional standardized perioperative antirheumatic drug discontinuation protocols that were uniformly applied to all RA patients receiving elective PLIF between 2015 and 2020. To achieve consistent and objective retrospective evaluation of historical medication data, we unified the judgment criteria for perioperative drug tapering and withdrawal by referencing the 2022 ACR/AAHKS Perioperative Medication Guidelines for Rheumatic Diseases together with domestic orthopedics-rheumatology expert consensus. This unified evaluation framework was tailored to address the inherent high infection and hemorrhage risks of spinal internal fixation surgery in RA patients receiving long-term immunosuppressive therapy.

### Radiological assessment

Preoperative lumbar x-ray, MRI and CT were used to assess the degree of spinal stenosis, bone destruction and spinal instability. Immediate postoperative radiographs were applied to verify spinal alignment and the position of internal implants.

Radiological parameters including disc height (DH), lumbosacral lordotic angle (LLA) and L1 vertebral HU value were measured by professional imaging software (18). DH was defined as the average value of anterior and posterior height of the target intervertebral space. LLA was measured as the Cobb angle between the superior endplate of L1 vertebra and the superior endplate of S1 vertebra (19) (Figure 1). All above radiological indicators were detected at three time points: preoperatively, immediately postoperatively and at the final follow-up.

**Figure 1:**
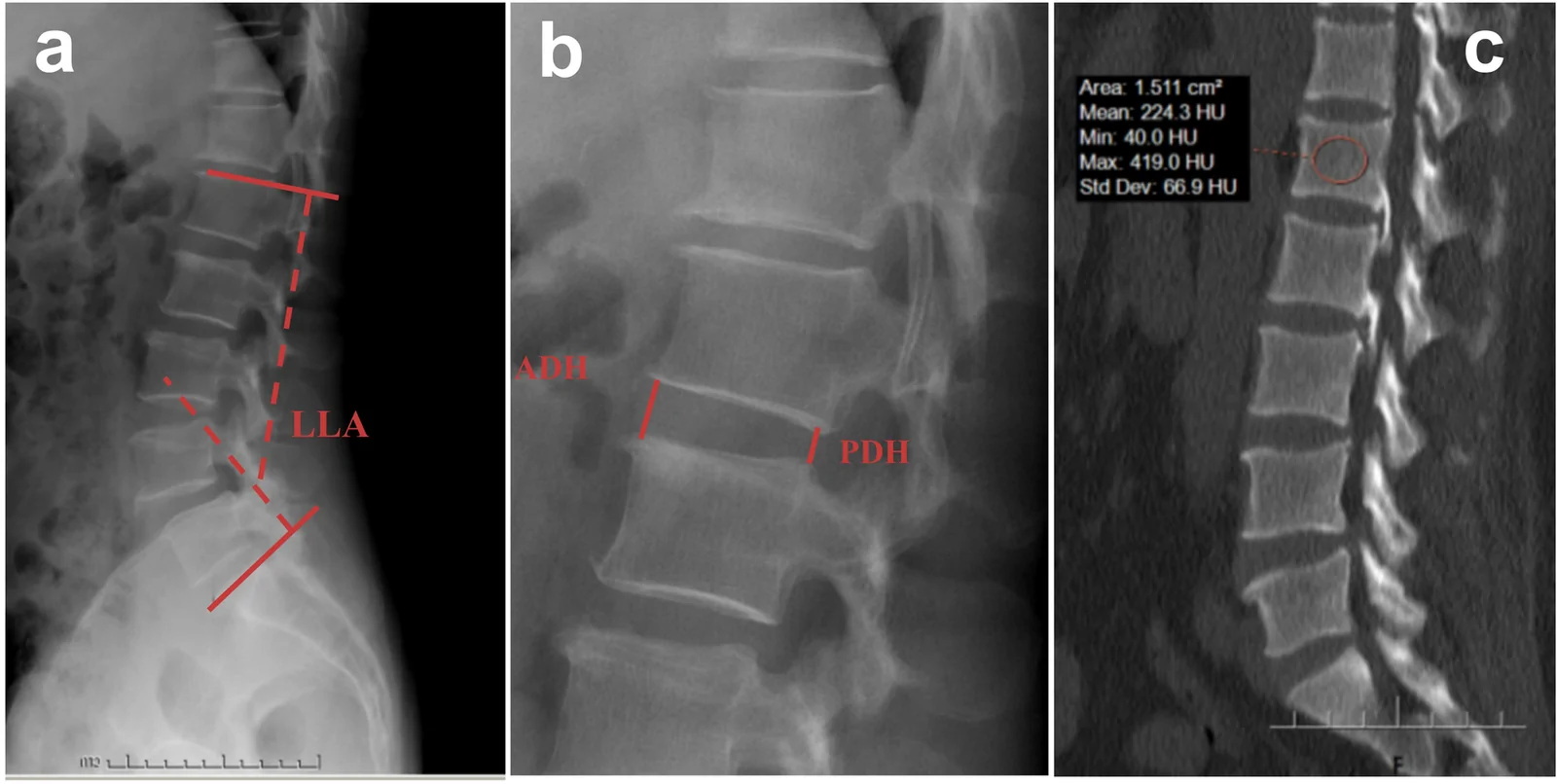
Representative images nof test parameters. **(a)** LLA was measured as the lines projected from upper endplate of L1 and upper endplate of S1. **(b)** DH was defined as the mean of the ADH and PDH of a given intervertebral space, DH = (ADH + PDH)/2. **(c)** Computed tomography scan illustrating the method of determining the L1-HU value with use of an elliptical region of interest. LLA, Lumbar lordosis angle; DH, Disk height; ADH, anterior disk heights; PDH, posterior disk heights.

The HU value of L1 vertebra was used to indirectly assess bone mineral density. According to the published standard, L1 HU value ≤ 100 was defined as osteoporosis (20). Radiological ASD was diagnosed when one of the following manifestations appeared: new anterior or posterior spondylolisthesis over 3 mm, adjacent segment disc height reduction over 3 mm, or anterior angular deformity greater than 5 degrees (21).

All radiological measurements were independently completed by two experienced orthopedic surgeons, and the average values were used for statistical analysis to minimize measurement bias (Figure 2).

**Figure 2:**
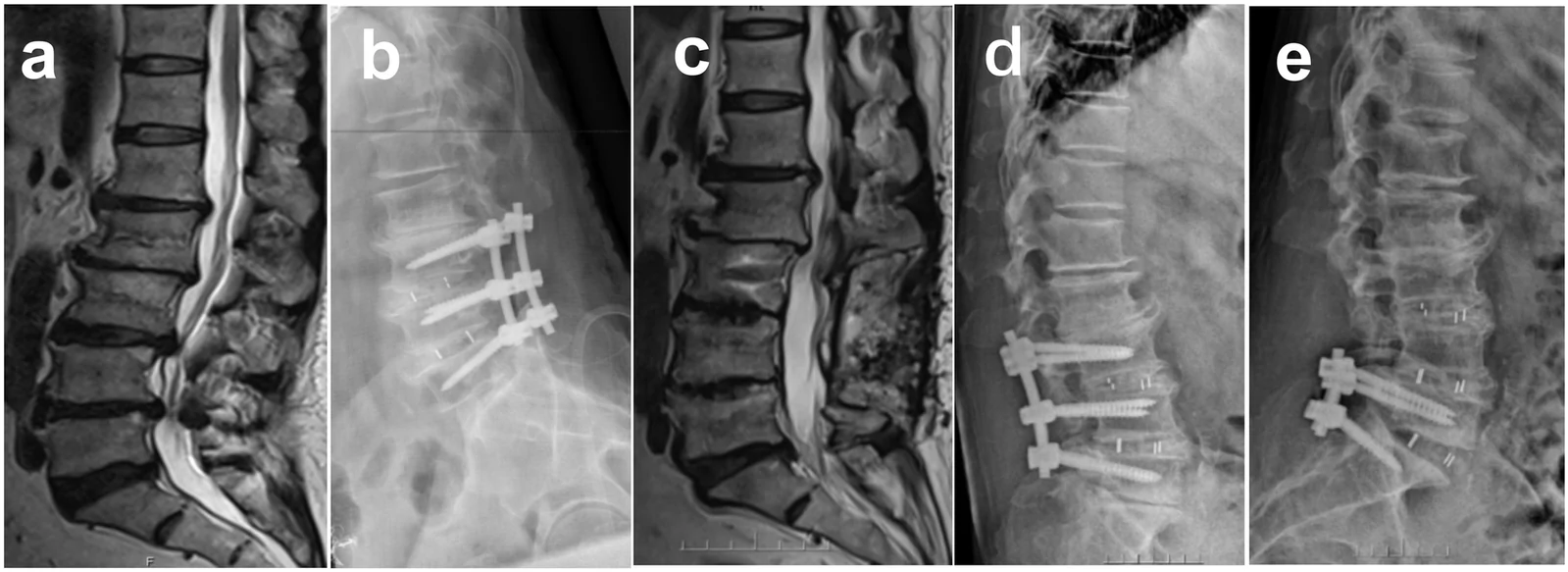
Revision surgery example of a 67-year-old man who underwent L3–L5 posterior lumbar interbody fusion. **(a)** Preoperative MRI reveal that L3-L5 lumbar disc herniation and lumbar spinal stenosis. **(b)** Postoperative x-ray show that the patient successfully received posterior lumbar interbody fusion surgery. **(c,d)** Review after 3 years, MRI and x-ray reveal that L5-S1 lumbar disc herniation and the height of the intervertebral space decreases. The patient reappeared with low back pain and leg pain. **(e)** The patient underwent a revision PLIF surgery by removing the previous pedicle screw.

### Statistical analysis

All statistical analyses were performed using SPSS 22.0 software. Continuous variables were presented as mean ± standard deviation; independent-samples *t*-test for normally distributed data, Mann–Whitney *U*-test for non-normal data. Categorical variables were reported as counts and proportions; intergroup comparisons adopted Chi-square test or Fisher's exact test for sparse cells. For binary postoperative complication endpoints, unadjusted odds ratios (OR), relative risks (RR) and corresponding 95%CI were calculated to quantify crude RA-associated risk. A *P*-value < 0.05 was defined as statistically significant.

## Results

### Baseline characteristics

All surgeries were completed successfully in all enrolled patients (Figure 2). The RA group consisted of 32 patients (5 males and 27 females), with the operative age of 61.09 ± 6.23 years and the BMI of 24.66 ± 3.58 kg/m^2^. The mean disease duration of RA was 16.09 ± 6.3 years (range, 8–30 years). According to Steinbroker classification, there were 8 cases of Grade I, 20 cases of Grade II and 4 cases of Grade III. Among RA patients, 20 received single-level fusion and 12 received double-level fusion.

The NRA group included 96 patients (15 males and 81 females), with the operative age of 58.63 ± 8.52 years and the BMI of 25.73 ± 3.25 kg/m^2^. There were 60 cases of single-level fusion and 36 cases of double-level fusion in this group. No significant differences in age, gender, BMI and fusion segments were observed between the two groups (Table 1).

**Table 1:** Clinical characteristics between RA patients and NRA patients.

Variables	RA (*n* = 32)	NRA (*n* = 96)	*P* value
Age (years)	61.09 ± 6.23	58.63 ± 8.52	0.08
Gender (male/female), *n*	5/27	15/81	
BMI (kg/m^2^)	24.66 ± 3.58	25.73 ± 3.25	0.12
Surgical level			
Single-level	20	60	
Double-level	12	36	
Steinbroker classification		/	/
Grade I	8		
Grade II	20		
Grade III	4		
Duration of RA (years)	16.09 ± 6.3	/	/

RA, rheumatoid arthritis; NRA, Non-RA.

### Perioperative indicators and clinical scores

For patients who underwent single-level fusion, the bed rest time was significantly longer in the RA group than that in the NRA group (*P* < 0.05). No significant differences were found in operative duration, intraoperative blood loss and hospital stay between the two groups. Preoperative C reactive protein (CRP) and white blood cell (WBC) counts were comparable between the two groups, while postoperative CRP levels were significantly higher in RA patients (*P* < 0.05) (Table 2).

**Table 2:** Perioperative parameters between RA patients and NRA patients.

Variables	RA (*n* = 32)	NRA (*n* = 96)	*P* value
Operative duration, min			
Single-level	146.75 ± 30.18	145.67 ± 33.16	0.61
Double-level	199.58 ± 64.89	196.39 ± 42.57	0.85
Intraoperative hemorrhage, mL			
Single-level	469 ± 160.29	442.6 ± 141.92	0.53
Double-level	762.5 ± 172.05	741.6 ± 263.36	0.75
Bed rest time, d			
Single-level	3.6 ± 1.05	3.03 ± 0.74	0.01
Double-level	3.83 ± 0.93	3.56 ± 0.77	0.46
Hospital stay, d			
Single-level	13.85 ± 5.41	11.53 ± 3.82	0.07
Double-level	19.66 ± 16.97	14.25 ± 5.89	0.18
C-reactive protein, μg/mL			
Preoperative	6.09 ± 10.4	3.02 ± 5.09	0.08
Postoperative	98.11 ± 42.79	64.99 ± 31.40	0.00
White blood cell, 10^9^/L			
Preoperative	5.57 ± 1.61	5.67 ± 1.71	0.78
Postoperative	9.23 ± 2.66	9.41 ± 2.52	0.74

RA, rheumatoid arthritis; NRA, Non-RA.

All patients experienced obvious pain reduction in the immediate postoperative period. However, distinct long-term pain trends were observed between groups: RA group presented continuously elevated back and leg VAS scores at final follow-up, while NRA group maintained sustained pain remission with decreased VAS values. Preoperative VAS and ODI scores showed no intergroup difference, but postoperative and final follow-up pain/function scores were significantly worse in RA patients (all *P* < 0.05) (Table 3).

**Table 3:** Clinical outcomes between RA patients and NRA patients.

Variables	RA (*n* = 32)	NRA (*n* = 96)	*P* value
VAS (back pain)			
Preoperative	6.44 ± 2.29	5.72 ± 2.11	0.08
Postoperative	2.62 ± 0.75	2.15 ± 0.73	0.00
Last follow-up	3.56 ± 1.66	1.69 ± 0.72	0.00
VAS (leg pain)			
Preoperative	6.5 ± 2.09	6.72 ± 2.0	0.54
Postoperative	2.46 ± 0.67	2.16 ± 0.79	0.01
Last follow-up	3.21 ± 1.43	1.52 ± 0.74	0.00
ODI			
Preoperative	67.55 ± 12.79	67.07 ± 12.12	0.45
Last follow-up	34.54 ± 15.40	18.20 ± 5.88	0.00

RA, rheumatoid arthritis; NRA, Non-RA; VAS, visual analog scale; ODI, Oswestry Disability Index.

### Radiological parameters and bone mass

The L1 HU value of the RA group was significantly lower than that of the NRA group (*P* < 0.05). Based on the diagnostic criterion of osteoporosis (HU ≤ 100), 8 patients (25.0%) in the RA group and 9 patients (9.38%) in the NRA group were diagnosed with osteoporosis, with a significant between-group difference (*P* < 0.05). DH and LLA showed no significant differences at preoperative, immediate postoperative and final follow-up time points between the two groups (Table 4).

**Table 4:** Radiological parameters between RA patients and NRA patients.

Variables	RA (*n* = 32)	NRA (*n* = 96)	*P* value
DH, mm			
Preoperative	7.78 ± 2.14	7.83 ± 2.27	0.91
Postoperative	10.43 ± 1.44	10.52 ± 1.76	0.785
Last follow-up	9.96 ± 1.34	10.37 ± 3.31	0.386
LLA			
Preoperative	42.53 ± 10.82	39.65 ± 13.54	0.278
Postoperative	46.13 ± 8.52	47.49 ± 10.65	0.512
Last follow-up	43.88 ± 11.16	45.91 ± 10.75	0.363
HU	117.71 ± 34.31	150.99 ± 45.62	0.00
Diagnostic for osteoporosis	8/32 (25%)	9/96 (9.38%)	0.035

RA, rheumatoid arthritis; NRA, Non-RA; DH, Disk height; LLA, lumbosacral lordotic angle; HU, Hounsfield Unit.

### Postoperative complications

The overall complication rate was markedly higher in the RA group (OR = 16.418, 95% CI 6.160–43.758, RR = 6.300, *P* < 0.001). Univariate risk analysis showed RA exposure correlated with significantly higher incidence of wound infection, ASD and revision surgery. All effect sizes reported herein are unadjusted without correction for potential confounding factors (Table 5).

**Table 5:** Complications between RA patients and NRA patients.

Variables	RA (*n* = 32) *n* (%)	NRA (*n* = 96) *n* (%)	*P*-value	Unadjusted OR	95%CI of OR	Unadjusted RR	95%CI of RR
Total complications	21 (65.6%)	10 (10.4%)	<0.00	16.41	6.16–43.76	6.30	3.33–11.92
Wound infection	4 (12.5%)	2 (2.1%)	0.03	6.71	1.17–38.60	6.00	1.15–31.23
VTE	3 (9.3%)	1 (1.04%)	0.05	9.83	0.984–98.13	9.00	0.97–83.49
Adjacent segment disease (ASD)	9 (28.1%)	5 (5.2%)	0.00	7.12	2.18–23.29	5.40	1.95–14.94
Revision surgery	5 (15.6%)	2 (2.1%)	0.01	8.70	1.60–47.39	7.50	1.53–36.79

All OR and RR values are unadjusted crude risk estimates without multivariate confounding correction.

RA, rheumatoid arthritis; NRA, Non-RA; VTE, Venous thromboembolism; ASD, Adjacent segment disease.

## Discussion

Univariate analysis in this matched retrospective cohort demonstrated that RA correlated with significantly elevated risks of multiple adverse outcomes after PLIF for lumbar degenerative diseases. RA patients suffered from poor wound healing and transient pain relief in the early postoperative period. During long-term follow-up, they presented worse functional recovery, higher ASD incidence and more revision surgeries. These findings supplement the clinical evidence regarding lumbar fusion surgery in RA patients.

Our study firstly found that RA patients obtained less satisfactory relief of low back and leg pain after surgery, which has not been fully reported in previous literature. The possible reasons are multifactorial. RA patients often suffer from multi-segment lumbar degeneration, and most of them are complicated with coexisting knee or hip synovitis. PLIF only targets the responsible lesion segment causing severe symptoms, while concomitant facet joint degeneration, paraspinal muscle atrophy and lower limb dysfunction cannot be corrected by this surgery, which persistently induce pain. In addition, the ODI scores of RA patients remained higher at long-term follow-up, which is consistent with the research results of Kang et al. (22). As a systemic inflammatory disease, RA continuously releases pro-inflammatory cytokines, disrupting the balance of bone resorption and formation and impairing bone remodeling. With aging, prolonged RA duration, progressive osteoporosis and long-term use of disease-modifying anti-rheumatic drugs (DMARDs) and immunosuppressants, joint degeneration is aggravated, eventually leading to combined lumbar and lower limb dysfunction. Therefore, clinicians should fully inform RA patients of the possible limited postoperative efficacy before surgery, and formulate targeted postoperative rehabilitation guidance.

In the present cohort, the intergroup difference in VTE incidence only reached borderline significance without statistical significance, which may be attributable to the limited sample size of the RA group. Even so, the crude VTE rate was numerically higher among RA patients, consistent with prior literature reporting that individuals with RA carry over two-fold elevated VTE risk relative to the general population (23). The pathogenesis of VTE in RA is closely related to systemic inflammation. Inflammatory cytokines such as interleukin-1beta and tumor necrosis factor-alpha (TNF-α) induce endothelial dysfunction, promote vascular infiltration of inflammatory monocytes and upregulate thrombogenic factors, thus leading to a hypercoagulable state (24–27). Activated platelets and abnormal coagulation function further accelerate thrombus formation (28). Besides, long-term use of non-steroidal anti-inflammatory drugs, glucocorticoids and JAK inhibitors in RA patients also increases the risk of VTE (28). In this study, RA patients had elevated postoperative CRP levels and longer bed rest time, which further increased the probability of VTE. Hence, standardized perioperative thromboprophylaxis is particularly important for RA patients undergoing lumbar fusion.

RA was also associated with a higher rate of postoperative wound infection, which has been validated in large-sample study (15). Active RA can reduce the diversity of circulating CD4^+^ T cells and shorten telomere length, weakening the ability of immune system to recognize and eliminate pathogens at the surgical site (13, 29). Long-term medication is another key factor: glucocorticoids inhibit the proliferation of monocytes and macrophages, while TNF-α inhibitors block the recruitment and activation of immune cells. Both types of drugs exert immunosuppressive effects and increase infection vulnerability (30, 31). The elevated postoperative CRP in the RA group in our study also reflected an excessive inflammatory response. For clinical practice, it is necessary to dynamically monitor inflammatory indicators preoperatively and postoperatively, and use prophylactic antibiotics rationally to reduce wound infection risk.

ASD is a common long-term complication after lumbar fusion and is the leading cause of revision surgery. The ASD incidence of 28.1% in our RA group was higher than that reported in previous study (22). In a study involving 137 patients, Alentado et al. (32) reported that the rate of symptomatic degeneration at an adjacent segment warranting re-operation was 13 (9%). The main risk factors for ASD are preoperative sagittal imbalance and multiple segmental fusion (33). Previous studies showed that spinal sagittal imbalance was relate to aging, loss of LL, and decreases in the quality and quantity of paravertebral muscle (34, 35). RA was an autoimmune disease that secreted inflammatory cytokines by activated T cells and synovial cells, such as TNF-α, interleukin-1 and interleukin-6 (21, 36). Inflammatory mediators lead to synovial inflammation, increased osteoclastic activity, decreased bone mineral density, and increased bone fragility. Meanwhile, pannus produced proteolytic enzymes leading to ligament and joint capsule relaxation or even rupture, resulting in lumbar instability, aggravating lumbar degeneration (37). Moreover, when and how to control the RA activity through medication is a controversy for RA patients, because various kinds of drugs may also influence bone metabolism and muscle, and increase the bone fragility and muscular atrophy (10). Seki et al. (21) reported that disease activity was associated with ASD, and controlling RA activity was crucial for preventing ASD after surgery. Therefore, consultation from the immune rheumatologist to evaluate the RA activity is important for RA patients if PLIF is required.

### Limitations and future perspectives

Several limitations of this study should be acknowledged. First, this is a single-center retrospective study, which is inherently limited by retrospective bias, and the generalizability of the conclusions needs to be verified by external cohorts. Second, the sample size of the RA group is relatively small, which is mainly attributed to the low incidence of lumbar surgery in RA patients. Third, all enrolled patients were of Asian ethnicity, so whether the results are applicable to other ethnic groups remains to be explored. Fourth, we did not conduct subgroup analysis based on RA duration, Steinbroker grade and different medication regimens, and the correlation between RA treatment strategies and surgical outcomes needs further research. Fifth, only univariate statistical analysis and unadjusted risk estimates were conducted in this study; multivariate regression models adjusting for osteoporosis, inflammatory status and age were not available. Further large-sample studies with multivariate adjustment are needed to validate independent predictive value of RA.

## Conclusion

RA increases the overall complication rate after PLIF for lumbar degenerative diseases. In the early postoperative stage, RA patients are prone to poor wound healing, persistent pain. In the long term, they have worse functional recovery, higher risks of ASD and revision surgery.

## Data Availability

The original contributions presented in the study are included in the article/Supplementary Material, further inquiries can be directed to the corresponding author/s.
